# Utilization of Waste Amine-Oxime (WAO) Resin to Generate Carbon by Microwave and Its Removal of Pb(II) in Water

**DOI:** 10.3390/toxics10090489

**Published:** 2022-08-23

**Authors:** Chunlin He, Yun Liu, Chunhui Zheng, Yanming Jiang, Yan Liao, Jiaxin Huang, Toyohisa Fujita, Yuezhou Wei, Shaojian Ma

**Affiliations:** 1 School of Resources, Environment and Materials, Guangxi University, Nanning 530004, China; 2 Guangxi Key Laboratory of Processing for Non-Ferrous Metal and Featured Materials, Guangxi University, Nanning 530004, China; 3 School of Nuclear Science and Technology, University of South China, Hengyang 421000, China; 4 School of Nuclear Science and Engineering, Shanghai Jiao Tong University, Shanghai 200240, China

**Keywords:** waste amine-oxime, Pb(II), adsorption, adsorbent, microwave

## Abstract

Utilising waste amine-oxime (WAO) resin through microwave semi-carbonization, a carbon adsorbent (CA) was obtained to remove Pb(II). After microwave treatment, the pore size of the skeleton structure, three-dimensional porous network, and lamellar pore structure of WAO was improved. The distribution coefficient (Kd) of Pb(II) onto CA is 620 mL/g, and the maximum adsorption capacity of Pb(II) is 82.67 mg/g after 20 min of WAO microwave treatment. The adsorption kinetics and adsorption isotherms conform to the quasi-second-order kinetic equation and Langmuir adsorption isotherm model, respectively. The surface of MT-WAO is negatively charged and the adsorption mechanism is mainly electrostatic interaction. Pb(II) elution in hydrochloric acid solution is more than 98%, and its recovery is high at 318 K and for 1 h.

## 1. Introduction

Lead (Pb) is one of the major heavy metal pollutants in the natural environment, which mainly comes from many industrial wastewaters such as metal mining, metal smelting, storage battery manufacturing, pulp, leather tanning and paper, etc. [1,2,3,4]. Once released, Pb can exist in the environment for a long time and is difficult to degrade; if it accumulates through the food chain, it can cause potential toxicity to plants, animals and even humans. Conventional Pb(II) contamination treatments include chemical precipitation, ion exchange, adsorption, membrane filtration, and electrodialysis [5,6,7]. Adsorption is widely considered a simple and low-cost method to remove metals from water [8,9,10].

China is the world’s key producer of ion exchange resin, which is mainly used in water treatment, hydrometallurgy, biopharmaceutical, food processing, and chemical industries. After prolonged use, pore collapse, shrinkage, reduction in the specific surface area, failure of functional groups, and generation of waste resin occur. For example, in the Guangxi branch of Chalco, amidoxime chelating resins utilized for gallium (Ga) adsorption from Bayer’s solution are discarded after reusing 50–60 times a month, and 225 tons of waste resin are produced and shelved each year [11,12]. Besides the Guangxi branch of Chalco, the waste amine-oxime is also produced in Henan, Shandxi, and Guizhou provinces in China, which used amine-oxime in the extraction of Ga. Currently, the central treatment methods for amidoxime chelating resins include stockpiling, landfill, curing and incineration. Incineration is more common, and the discarded resin is sent to solid waste handling companies for incineration at about 2000 RMB/ton [12]. After incineration, many polluting gases, such as CO_2_, CO, SO_2_, NO and NO_2_, are produced, which is not conducive to controlling carbon emissions. In addition, the storage and landfill of waste resin occupy a larger land area, which neither produces economic benefits nor meets the requirements of environmental protection.

Much research has been carried out on the recycling of waste resin. Long et al. [13] obtained a carbon adsorbent by carbonization and activation of the waste polysulfonated cation exchange resin. The activated carbon exhibited adsorption performance for naphthalene. Shi et al. [14] impregnated ferric nitrate into waste resin (D001), and then the waste resin was carbonized and activated to obtain C/Fe composite magnetic adsorption material. Shi et al. [15] used waste polystyrene ion-exchange resin to prepare activated carbon under nitrogen at 450 °C for 30 min and then activated at 800 °C; it exhibited excellent adsorption performance for naphthene and other polycyclic aromatic hydrocarbons. Wei et al. [16,17,18,19] used waste ion-exchange resin to prepare activated carbon for CO_2_ adsorption, with a specific surface area of 400–900 cm^2^/g. Bratek et al. [20,21,22] studied the carbonization of waste ion exchange resin at 600~850 °C to prepare an activated carbon adsorbent, which exhibited excellent adsorption performance for toluene, oil, copper, and lead ions in wastewater. Gun’ko et al. [23] investigated 5 kinds of waste resins to obtain activated carbon with a specific surface area of 200~600 m^2^/g, and they could effectively remove formaldehyde (0.33 cm^3^/g). Miura et al. [24] conducted a study on the preparation of porous activated carbon by carbonization of two waste resins at 500~900 °C, and the results showed that adding different metal ions could change the pore size distribution.

In summary, for the recycling of waste resin, many works focus on the preparation of activated carbon by using ion-exchanged resin. Most of the activated carbon consumes large energy, and activated carbon becomes powder, not spherical. The recycling of waste amine oxime resin has not been studied systematically. Amine oxime resin is different from ion exchange resins; it receives strong acid and strong base impact during the extraction of Ga. The internal pores of WAO resin are not developed, which exhibits collapse and shrinkage. In this study, for waste utilization, the waste amine oxime (WAO) resin was treated by microwave semi-carbonization (to improve the pores) to obtain a granular adsorbent to remove Pb(II). After microwave treatment, the resin also retains the sphere. In addition, the effects of microwave treatment time, adsorption time, and co-existing ions in the solution on the adsorption of Pb(II), as well as the effects of time and temperature on elution, were investigated; this work could also provide a reference for other waste resins.

## 2. Materials and Methods

### 2.1. Materials

The waste amine oxime (WAO) resin, with a particle diameter of 0.2–0.6 mm, was obtained from the Guangxi branch of Chalco. Reagent grade Pb(NO_3_)_2_ (Aladdin) and deionized water were used in all the experiments. The WAO resin was rinsed with hydrochloric acid (analytical-grade) and ultrapure water to remove the organic and inorganic impurities.

### 2.2. Characterization Methods

The surface morphology and corresponding elemental distribution were analyzed by scanning electron microscopy with energy dispersive spectroscopy (SEM-EDS, Phenom Pro 800-07334, Eindhoven, The Netherlands). The adsorption mechanism was studied by X-ray photoelectron spectroscopy (XPS, ESCALAB 250XI+, Waltham, MA, USA) equipped with a monochromatized Al-Kα X-ray source and Fourier-transform infrared spectroscopy (FT-IR, THS-108, USA). The C, N, O and H contents of FAO and WAO were estimated using an elemental analyzer (Vario EL cube, Han, Germany). The zeta potential of the suspension (100 mg/L) containing nonmetallic particles of the MT-WAO (200 mesh) was measured by using a high-sensitivity zeta potential analyzer (NanoBrook Omni, New York City, NY, USA) to explore the surface charge as a function of pH. Thermogravimetry and differential thermal analysis (TG-DTA) were carried out in a nitrogen atmosphere with a heating rate of 10 °C/min.

### 2.3. Microwave Semi-Carbonisation Methods

Due to the collapse and shrinkage, the internal pores of WAO resin are not developed. Therefore, a household microwave oven (EV025LC7-NR, Midea) with a microwave output power of 1000 W was used to improve the pores. Microwave heating is fundamentally different from conventional heating methods, as it uses microwave interactions with materials at molecular or atomic levels to generate heat. Microwave processing involves penetrating radiation, controllable electric field distribution, rapid heating, selective heating of materials through differential absorption and self-limiting reaction. Therefore, 10 g WAO resin was introduced into a quartz cup, the mouth of the cup was completely sealed with paraffin film, and kept in the microwave oven for different times (2–20 min) to obtain a new absorbent (MT-WAO).

### 2.4. Adsorption Experiments

The static adsorption experiments were performed in a thermostatic water bath to investigate the influence of various experimental parameters on the adsorption capacity. The solution concentration was analyzed using a standard curve method by ICP-AES (Inductively coupled plasma-atomic emission spectrometry, Shimadzu ICPS-7510, Kyoto, Japan). The speed of the stirrer in the water bath oscillator is 140 rpm. The relevant experimental results were calculated using Equations (1) and (2)

*Q* = (*C*_0_ − *C*) × (*V*/*m*) (1)

*K_d_* = [(*C*_0_ − *Cs*)/*Cs*] × (*V*/*m*) (2)

where *Q* (mg/g) and *K_d_* (mL/g) are the adsorption capacity at the equilibrium state and the distribution coefficient, respectively. *C_o_* (mg/L) and *C* (mg/L) denote metal ions’ initial and equilibrium concentrations, respectively. *C_s_* represents the metal concentration in the aqueous phase after adsorption. *V* (L) is the volume of the aqueous phase, and *m* (g) represents the mass of WAO resin.

## 3. Results

### 3.1. Characteristics of WAO

**Figure 1:**
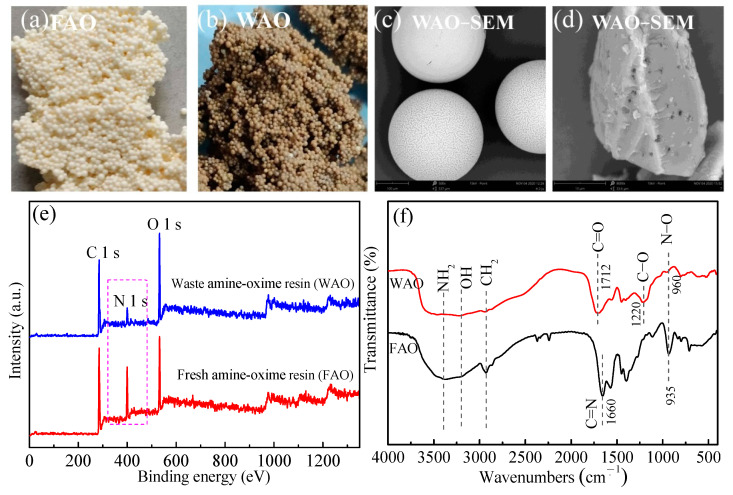
The structural features and elements of fresh and waste amine oxime resin: (**a**) fresh amine-oxime resin (FAO), (**b**) waste amine-oxime resin (WAO), (**c**,**d**) SEM of WAO, (**e**) XPS of WAO and FAO, (**f**) FT-IR of WAO and FAO.

The structural features and elements of fresh amine oxime resin (FAO) and WAO are presented in Figure 1. WAO exhibits a compact spherical particle with shrinkage and pore clog (Figure 1d). Compared with FAO, N in WAO is lower, and O is higher (Table 1). C and H contents of the resin changed slightly, while O content increased from 57.86% to 88.29%, and the N content decreased from 23.96% to 8.24%. Based on Figure 1e, the intensity of the N 1 s peak of WAO resin is significantly weakened. In comparison, the intensity of the O 1 s peak is significantly enhanced, consistent with the results shown in Table 1.

Figure 1f shows the peaks of FAO at 1650 cm^−1^, 1109 cm^−1^ and 935 cm^−1^, which correspond to tensile vibrations of C=N, C-N and N-O bonds [25]; however, in the case of WAO resin, these peaks shift to higher wavenumbers, and their intensities decrease significantly; furthermore, two new and extremely sharp characteristic peaks appear at 1697 cm^−1^ and 1195 cm^−1^, corresponding to the tensile vibrations of C=O and C-O bonds in the carboxyl group [26,27], attributed to the oxidation of amine oxime group into carboxyl group [28].

**Figure 2:**
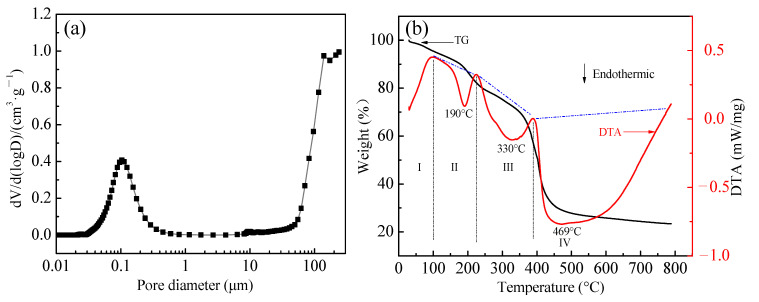
(**a**) Differential pore volume distribution of pores with different diameters in the WAO resin (**b**) TG and DTA curves of WAO resin.

WAO’s micropore and mesopore structures were almost completely blocked when the BET and BJH models were used to analyze the specific surface area and pore volume. Therefore, WAO’s pore sizes were measured by Mercury Porosimeter (Micrometric Autopore 9620, Sarasota, Florida, USA), as shown in Figure 2a.

Figure 2a shows that the total porosity of WAO resin is 49.29%, the intra-particle porosity is 34.56%, the interparticle porosity is 14.73%, and the pore volume is 0.65 cm^3^/g, and the specific pore surface area is 8.49 m^2^/g. The pore size distribution of WAO resin presents bimodal characteristics, and its pore volume and pore-specific surface area concentrate in the macro pores. The pore size of the main peak ranges from 8.1 μm to 96.2 μm, accounting for 10.19% of the total pore volume. The pore size of the small peak ranges from 0.02 μm to 0.3 μm, accounting for 28.63% of the total pore volume. The distribution of the largest pore size ranges from 96.2 to 238.4 μm, with a pore volume ratio of 59.5%; however, it is not in the effective pore size test range (7 nm–100 μm), which may be due to the interference of interparticle voids under low-pressure conditions, resulting in a large pore volume ratio.

The stability of WAO resin was characterized by TG-DTA, as shown in Figure 2b. When the temperature increases to 100 °C (step I), the mass loss is 4.6%. And then, a downward endotherm peak appears at 190 °C in the DTA curve (step II); this may be due to the volatilization of hydrated water. The decomposition at the temperature of 220–390 °C (step III) is attributed to the depolymerization of polystyrene–divinylbenzene structures present in the resin. Finally, the mass of WAO resin rapidly reduced by nearly 24% over the temperature range of 390–600 °C (step IV), possibly due to polystyrene and divinylbenzene structure degradation. Therefore, to obtain certain mechanical strength and spherical shape absorbent, the temperature shall be controlled below 220 °C when a microwave is applied to waste resin.

### 3.2. Characteristics of MT-WAO

**Figure 3:**
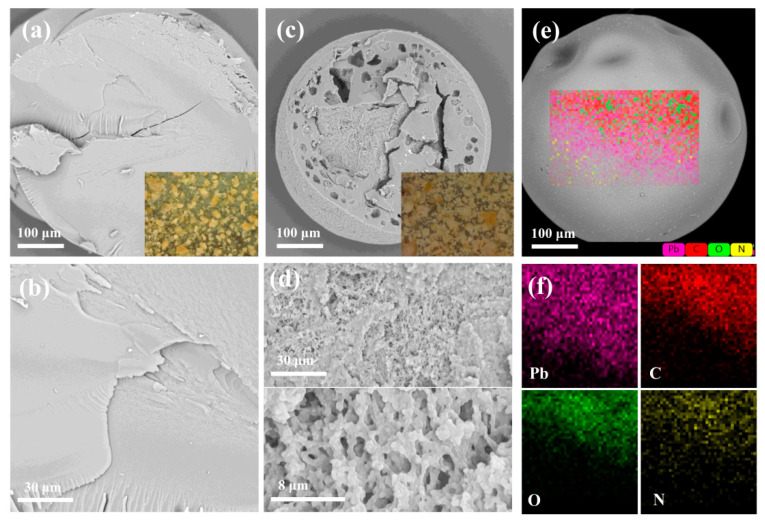
SEM images of (**a**,**b**)WAO resin (**c**,**d**) MT-WAO-resin (**e**,**f**) EDS of MT-WAO resin after adsorbing Pb(II).

The microstructure of WAO resin before and after microwave treatment was characterized by SEM-EDS, and the obtained images are shown in Figure 3.

No pores could be observed in the WAO resin (Figure 3a, magnified 500 times). The cross-section is rough with uneven holes (Figure 3c) after microwave treatment. When the waste resin was heated by a high-power microwave, the temperature of waste resin was quickly increased in a short time, especially the part inside. Then, the adsorbed water and hydrated water were heated up quickly and became gaseous, and then high pressure occurred inside of waste resin, making pores develop. As shown in Figure 3c, the resin also retains its sphere. MT-WAO resin presents a lamellar stack structure and a connected three-dimensional porous network structure (Figure 3d), indicating that the pore structure of WAO resin was improved after microwave heating; it can be observed from Figure 3e that MT-WAO resin still presents a regular spherical shape after adsorbing Pb(II). The adsorbed Pb(II) is evenly distributed on the surface of MT-WAO resin (Figure 3f).

**Figure 4:**
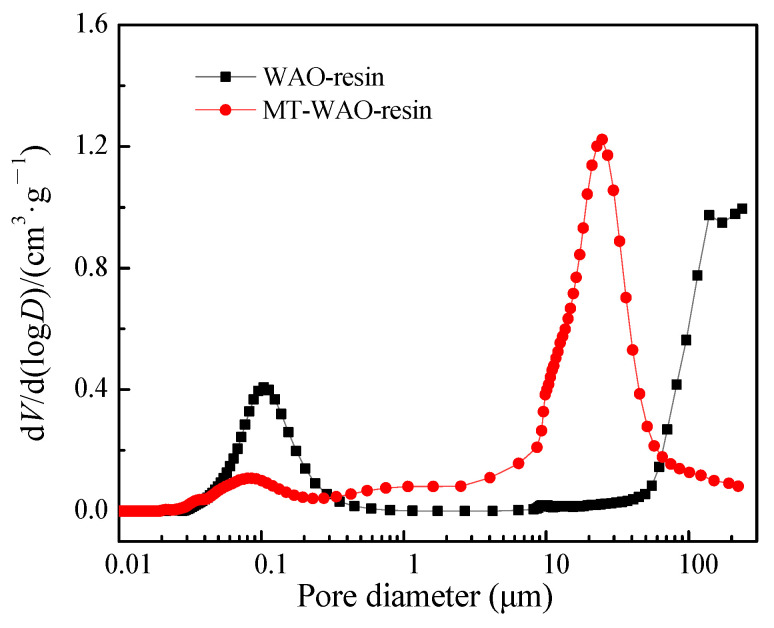
Differential pore volume distribution of pores with different diameters of MT-WAO resin.

For MT-WAO resin, the total porosity is 55.37%, the intra-particle porosity is 46.31%, the interparticle porosity is 9.06%, the pore volume is 0.84 cm^3^/g, and the pore-specific surface area is 3.89 m^2^/g (Figure 4, measurements obtained using mercury porosimeter). The pore size distribution of MT-WAO resin presents bimodal characteristics, and its pore volume and pore-specific surface area are mainly distributed in the macroporous range. The main peak appears in the range of 4.0–57.4 μm, and the total pore volume is 72.3%. The pore size of the small peak ranges from 0.02 μm to 0.3 μm, with a total pore volume of 3.12%. The transitional pore size between the two peaks ranges from 0.3 μm to 4.0 μm, with a total pore volume of 10.25%.

By comparing the pore structure of WAO resin, it is found that the pore volume of the pore size of less than 1 μm decreases. Microwave treatment causes the pores (<1 μm) to develop into 4.0–57.4 μm super pores. The total porosity and internal porosity of MT-WAO resin increase slightly, making WAO resin’s pore structure more developed.

### 3.3. Adsorption

#### 3.3.1. Effect of Microwave Treatment Time

Microwave heating was applied to treat WAO for different times (2–20 min). Then, a batch of MT-WAO was obtained for the adsorption test. The adsorption test conditions were: pH 4.0, solid-liquid ratio 250 g/mL, initial metal ion concentration 100 mg/L, temperature 298 K, and time 7 h.

**Figure 5:**
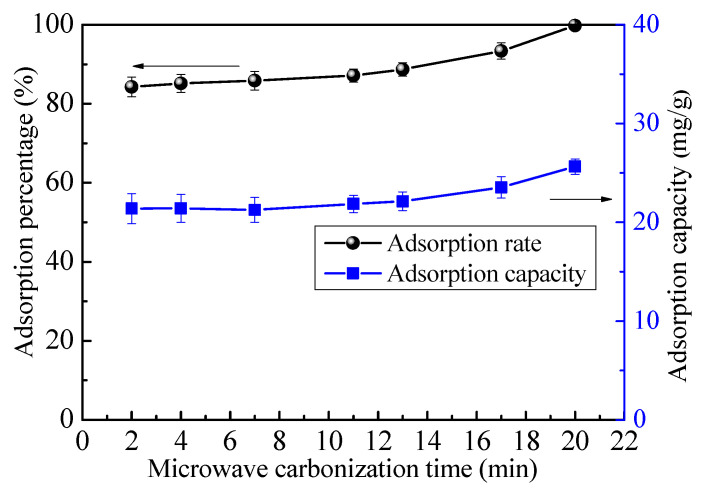
Effect of microwave treatment time on the adsorption of Pb(II).

As Figure 5 shows, with an increase in the microwave treatment time, the adsorption capacity and efficiency of MT-WAO resin for Pb(II) remain the same at first and then gradually increase. When the microwave treatment time is 20 min, the adsorption capacity and Pb(II) adsorption rate are 24.9 mg/g and 99.7%, respectively. Therefore, the optimal contact time of WAO resin treated by microwave is 20 min which was considered in the subsequent tests.

#### 3.3.2. Effect of pH

**Figure 6:**
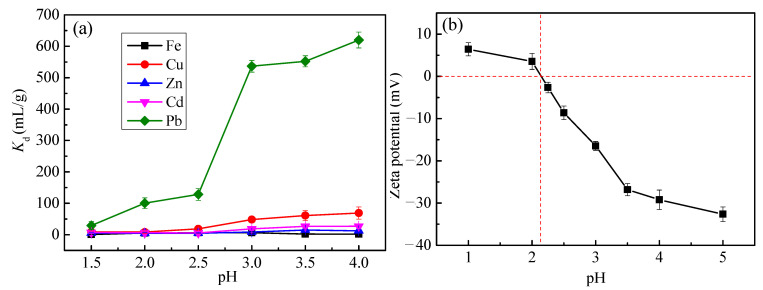
(**a**) Effect of pH on the adsorption of Pb(II) onto MT-WAO resin, (**b**) zeta potential of MT-WAO.

The effect of initial pH on adsorption is shown in Figure 6a. The concentration of ions is 100 mg/L. To prevent the formation of hydroxide precipitation, the pH range was controlled at 1.0–4.0, the liquid-solid ratio was 250 mL/g, the temperature was 298 K, and the time was 7 h.

When the pH value is between 1.5 and 2.5, the *Kd* of Cu(II), Zn(II), Cd(II) and Fe(II) metal ions is small, indicating that the ability of MT-WAO resin for adsorption of these metal is weak (Figure 6a). *Kd* of Pb (II) increases with the increase of pH, which means that the ability of MT-WAO resin for Pb(II) adsorption is enhanced as pH increases. When the pH is above 3.0, MT-WAO resin exhibits better adsorption for Pb(II), and the maximum *Kd* of Pb(II) reaches 620 mL/g.

Figure 6b shows that the zeta potential of the MT-WAO decreases as pH increases, and the point of zero charge pH is 2.1. In the pH range 1.0–2.1, the value of the zeta potential is positive, as the pH value exceeds 2.1, the MT-WAO possesses an overall negative surface charge, which makes it to attract the positively charged metal ions in the pores by electrostatic interaction. Based on this, the subsequent tests were carried out at pH 4.0.

#### 3.3.3. Effect of Time and Adsorption Kinetics

**Figure 7:**
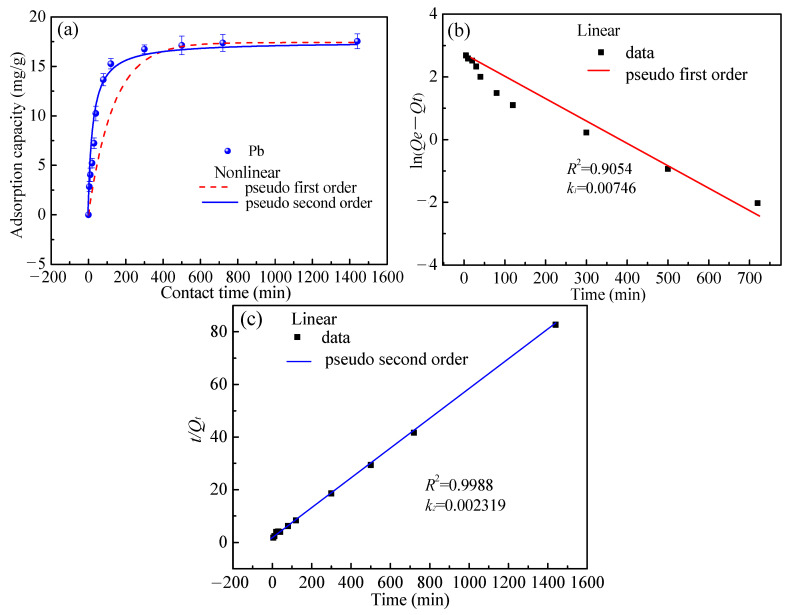
The adsorption kinetics. (**a**) effect of time on adsorption; (**b**) the fitting of pseudo first order; (**c**) the fitting of pseudo second order.

The influence of time on the adsorption of Pb(II) was investigated in the range of 5 min to 24 h (Figure 7). The temperature was 298 K, the pH was 4, the liquid-solid ratio was 250 mL/g, and the initial metal ion concentration was 100 mg/L. During 5–300 min of adsorption, the adsorption efficiency of Pb(II) increased with time and remained unchanged after it was extended to 300 min. When the adsorption equilibrium time is 300 min, the equilibrium adsorption capacity of Pb(II) reaches 17.4 mg/g.

To analyze the reaction kinetics, the pseudo-first-order-kinetic and pseudo-second-order-kinetic [11,29,30] models were applied, as presented in Equations (3) to (6), respectively.

(3) $$\text{Lagergren}’s\;\text{pseudo-first-order}:\;Q_{t}=Q_{e}(1-e^{-k_{1}t})$$

(4) $$\text{Linear}\;\text{equation}:\;\text{ln}(Q_{e}-Q_{t})=\ln Q_{e}-k_{1}t$$

(5) $$\text{Ho}’s\;\text{pseudo-second-order}:\;Q_{t}=\frac{Q_{e}^{2}k_{2}t}{1+Q_{e}k_{2}t}$$

(6) $$\text{Linear}\;\text{equation}:\;\frac{t}{Q_{t}}=\frac{1}{k_{2}Q_{e}^{2}}+\frac{1}{Q_{e}}$$

where *k*_1_ (min^−1^) and *k*_2_ (g/mg/min) are the kinetic rate constants. *Q_t_* and *Q_e_* (mg/g) represent the extent of metal ions adsorbed per unit adsorbent at time *t* and equilibrium state, respectively.

The detailed parameters for the two models are depicted in (Figure 7b,c); it can be concluded that the adsorption of Pb^2+^ obeys the pseudo-second-order kinetic model owing to the following aspects: the correlation coefficients (*R*^2^) obtained for the pseudo-second-order model are higher and near to 1.

#### 3.3.4. Effect of Temperature and Adsorption Isotherm

**Figure 8:**
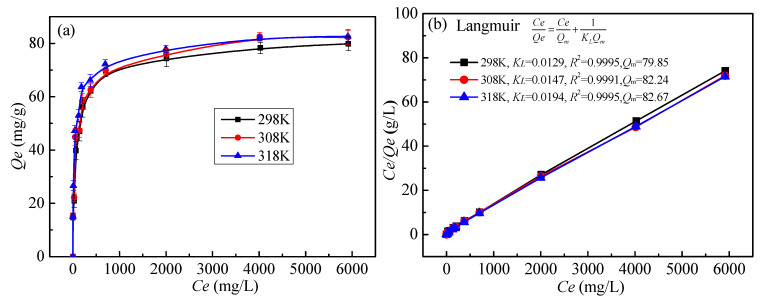

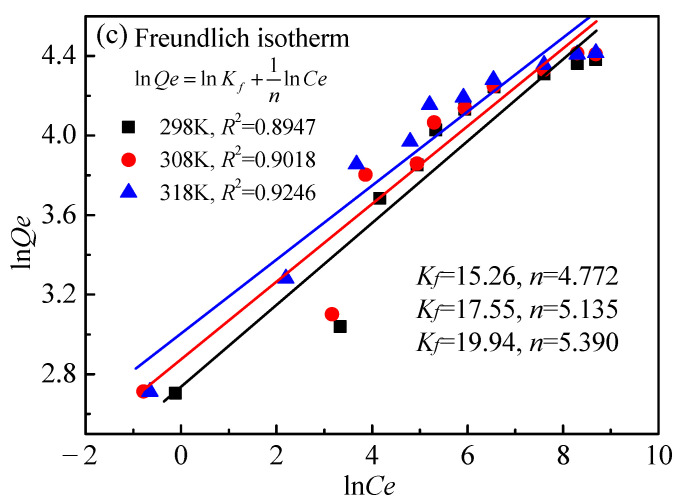
The adsorption isotherms of Pb(II) on the MT-WAO resin: (**a**) the influence of the initial concentration of Pb(II) on the adsorption, (**b**) the fitting of Langmuir model, (**c**) the fitting of Freundlich model.

The influence of the initial concentration of Pb(II) on the adsorption isotherm was investigated at room temperature (Figure 8a). The test conditions were: the liquid to solid ratio of 300 mL/g, pH 4.0, temperature 298 K, time 7 h, and initial metal ion concentration 50–6000 mg/L. As shown in Figure 8a, the adsorption capacity of MT-WAO resin for Pb(II) increases first; it then remains the same with an increase in the initial concentration of metal ions. When the initial concentration was 1000 mg/L, it reached the adsorption equilibrium, and the actual saturated adsorption capacity was 82.67 mg/g at 318 K.

The adsorption isotherms can be fitted by Langmuir [31] and Freundlich [32] isotherm models expressed as Equations (7) and (8), respectively (Figure 8a,c), and the fitting parameters are shown in Table 2. The Langmuir model (Figure 8b) is more consistent with the adsorption data (*R*^2^ = 0.99), indicating that Pb(II) is adsorbed on MT-WAO resin as a monolayer and uniformly; this suggests that the metal ion adsorption on the waste resin surface is an entirely homogeneous process and follows a monolayer adsorption mechanism. The adsorption capacity of MT-WAO resin for Pb(II) reaches 82.67 mg/g at 318 K. Therefore, MT-WAO resin is an excellent adsorbent and can effectively adsorb Pb(II) from the solution.

(7) $$\mathrm{Langmuir}\;\mathrm{model}:\;\frac{C_{e}}{Q_{e}}=\frac{C_{e}}{Q_{m}}+\frac{1}{K_{L}Q_{m}}$$

(8) $$\mathrm{Freundlich}\;\mathrm{model}:\;\ln Q_{e}=\ln K_{f}+\frac{1}{n}\ln C_{e}$$

where *Q_e_* and *Q_m_* represent the equilibrium and maximum adsorption capacities (mg/g), respectively. *C_e_* is the equilibrium metal ion concentration (mg/L), *K_L_* and *K_f_* are the Langmuir and Freundlich constants separately, and 1/*n* is the Freundlich heterogeneity factor.

Thermo-dynamic parameters [33] were calculated for this system by using Equations (9) and (10).

(9) $$\mathrm{Langmuir}\;\mathrm{model}:\;\ln K_{d}=\frac{\Delta S}{R}-\frac{\Delta H}{RT}$$

(10) Freundlich model: ΔG=ΔH−TΔS

where Δ*H*, Δ*S*, and *T* are the enthalpy, entropy, and temperature in kelvin, respectively, and *R* (8.314J/(mol·K)) is the gas constant.

The values of enthalpy (Δ*H*) and entropy (Δ*S*) were obtained from the slope and intercept of ln*K_d_* vs. 1/*T* plots, which were calculated by a curve-fitting program. The values of the thermodynamic parameters for the sorption of Pb(II) on MT-WAO are given in Table 3. The positive value of enthalpy change Δ*H*, shows that the adsorption of Pb(II) is endothermic. The numerical value of Δ*G* decreases with the increase in temperature, indicating that the reaction is spontaneous and more favorable at a higher temperature.

In this study, the low-cost MT-WAO exhibits good extractive properties for Pb(II) in terms of the adsorption capacity as compared to that of the adsorbents reported previously (Table 4); it reveals that the MT-WAO is a good adsorbent for the treatment of Pb(II) from the waste aqueous.

### 3.4. Adsorption Mechanism

**Figure 9:**
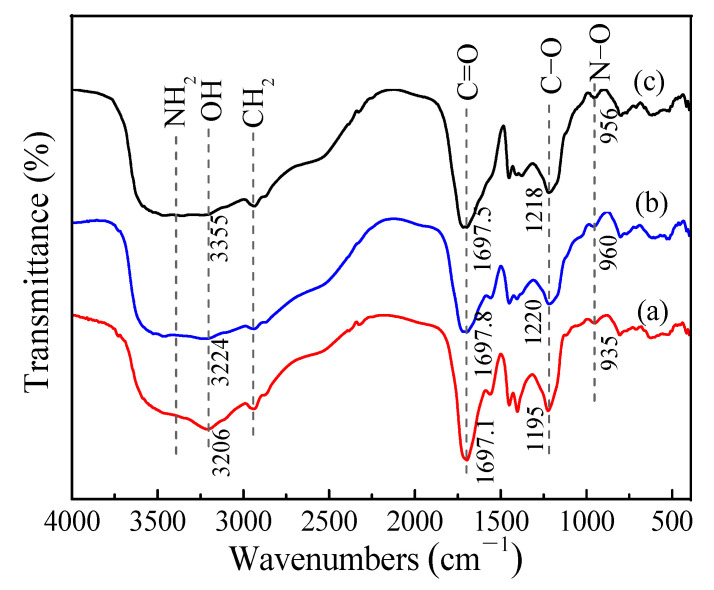
FT-IR spectra of (**a**) WAO and MT-WAO resins (**b**) before and (**c**) after the adsorption of Pb(II).

**Figure 10:**
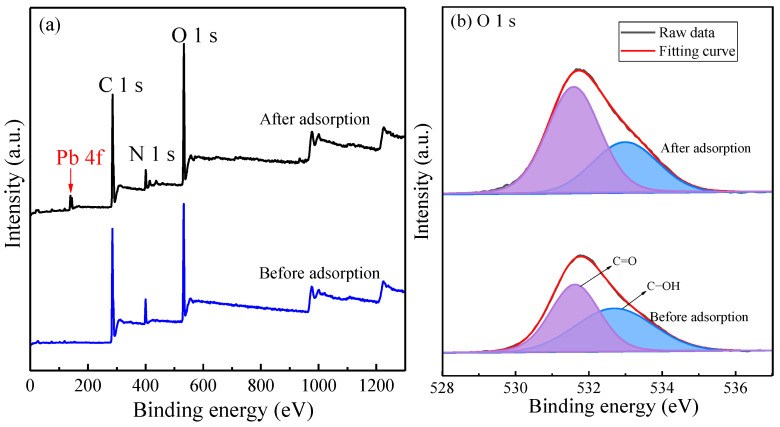
XPS (**a**) survey spectra and (**b**) regions of O 1 s of MT-WAO resin before and after the adsorption of Pb(II).

WAO and MT-WAO resins before and after the adsorption of Pb(II) were characterized by FT-IR and XPS, as shown in Figure 9 and Figure 10, respectively. The FTIR spectrum of MT-WAO resin, as shown in Figure 9, illustrates that the peak with high intensity can still be observed at wavelengths 1697 cm^−1^ and 1220 cm^−1^, corresponding to the tensile vibrations of C=O and C−O in the carboxyl group [38]; this indicates that −COOH functional group still exists in MT-WAO resin; however, by comparing the infrared spectra of MT-WAO resin before and after the adsorption of Pb(II), the peaks of MT-WAO resin at 1697 cm^−1^ and 1220 cm^−1^ are not shifted significantly after the adsorption of Pb(II).

Figure 10a shows the characteristic peak corresponding to Pb 4f at the binding energy of 138.8 eV in the XPS spectra after the adsorption of Pb(II). Figure 10b exhibits the high-resolution XPS spectra of the O 1 s region. O 1 s peaks of MT-WAO resin before adsorption are fitted into 531.63 eV and 532.69 eV peaks, corresponding to C=O and C-OH. After Pb(II) adsorption, the corresponding binding energies shift to 531.58 eV and 532.99 eV. The characteristic peak C=O and C-OH are almost unchanged, indicating that the adsorption mechanism is not in coordination.

**Figure 11:**
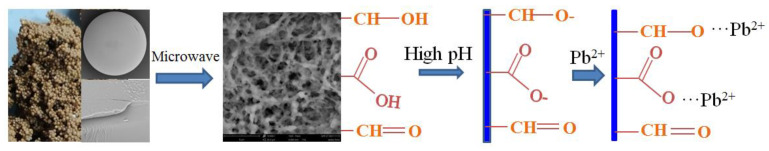
The schematic diagram of the possible adsorption mechanism of the MT-WAO.

FT-IR shows that the surface oxygen-containing groups of MT-WAO are mainly -OH, =C=O and –COOH; these groups may react with H^+^ to form –OH_2_^+^, =COOH_2_^+^ and –C=OH^+^, resulting in the surface of MT-WAO being positively charged. When pH increases, it will reduce the number of positive ions on the surface of MT-WAO. When pH exceeds the point of zero charge pH is 2.1, the surface of MT-WAO will be negatively charged. Therefore, the adsorption mechanism is electrostatic interaction. The schematic diagram of the possible adsorption mechanism of the MT-WAO is shown in Figure 11.

### 3.5. Elution

In order to explore the recovery effect of Pb(II), MT-WAO resin loaded with Pb(II) under optimal adsorption conditions was used as the raw sample for elution and hydrochloric acid solution of 1 mol/L was selected as the desorbing agent. The effects of time and temperature on Pb(II) resolution were investigated, as shown in Figure 11. The desorbed conditions were: 1 mol/L hydrochloric acid solution and the liquid-solid ratio was 50 mL/g.

**Figure 12:**
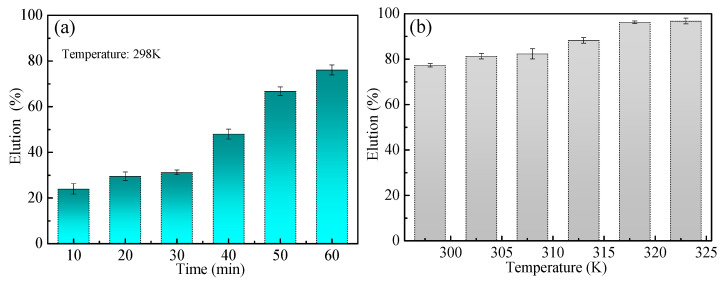
Variation of elution percentage of Pb(II) with (**a**) time and (**b**) temperature.

Figure 12a shows that the elution rate of Pb(II) increases gradually with time, which reaches more than 75% when the time reaches 1 h. The elution time greatly influences the elution of Pb(II). Figure 12b exhibits that the elution rate of Pb(II) increases gradually with temperature. When the temperature increased to 318 K during 1 h, 1 mol/L hydrochloric acid solution was sufficient to completely elute the Pb(II) adsorbed from MT-WAO resin with an elution rate of 98%.

## 4. Conclusions

In this investigation, after subjecting WAO-resin to microwave, chaotic pore structure and the original dense structure changed into lamellar stack structure or interconnected three-dimensional porous network structure. WT-WAO resin exhibited good selectivity towards adsorbing Pb(II) (Kd 620 mL/g). The optimal microwave treatment time and optimal pH are 20 min and 4, respectively. The adsorption reached equilibrium at 300 min. The maximum adsorption capacity of Pb(II) reached 82.67 mg/g at 318 K. The adsorption kinetics and adsorption isotherms of WT-WAO resin for Pb(II) conform to the quasi-second-order kinetic equation and Langmuir adsorption isotherm model, respectively. The adsorption behavior of WT-WAO-resin for Pb(II) is homogeneous and adsorbed in a monolayer. The surface of MT-WAO is negatively charged and the adsorption mechanism is mainly electrostatic interaction. When the temperature increased to 318 K during 1 h, 1 mol/L hydrochloric acid solution was sufficient to completely elute the Pb(II) adsorbed from MT-WAO resin with an elution rate of 98%.

## Figures and Tables

**Table 1:** C, N, O and H contents in the FAO and WAO.

Element (%)	FAO	WAO
C	46.07	46.04
O	57.86	88.29
N	23.96	8.240
H	3.047	5.373

**Table 2:** The fitting parameters of the adsorption isotherms for the Langmuir and Freundlich models.

Metal Ion	*T*(K)	Langmuir Isotherm Model			Freundlich Isotherm Model		
		*Q_m_* (mg/g)	*K_L_*	*R* ^2^	*K_f_*	*n*	*R* ^2^
	298	79.85	0.0129	0.9995	15.26	4.772	0.8947
Pb(II)	308	82.24	0.0147	0.9991	17.5	5.135	0.9018
	318	82.67	0.0194	0.9992	19.94	5.390	0.9246

**Table 3:** Thermodynamic parameters for the adsorption of Pb^2+^ on MT-WAO resin.

Concentration of Pb^2+^ (mg/g)	Δ*H* (J/mol)	Δ*S* (J/mol)	Δ*G* (KJ/mol)		
			298 K	309 K	318 K
50	21.15	94.16	−6.21	−6.42	−6.63
100	54.01	177.48	−52.83	−54.61	−56.38
200	26.07	83.73	−24.93	−25.76	−26.60
300	10.46	25.66	−7.64	−7.89	−8.15
400	10.01	22.55	−6.71	−6.94	−7.16

**Table 4:** Comparison of Pb^2+^ and Cu^2+^ adsorption performance of the MT-WAO with other adsorbents.

Adsorbents	Adsorption Capacities (mg/g)	References
	Pb^2+^	
MT-WAO	82.67	This work
Sago waste	109.7	[34]
Aminated polyacrylonitrile fibers	76.1	[35]
Lignocellulosic biomaterial	62.1	[36]
Activated carbon	30.4	[37]
Guanyl-modified cellulose	52.0	[38]
Weak acidic cation resin	58.1	[39]
Pigeon peas hulls	20.8	[40]
Chars from Prosopis Africana	45.3	[41]

## Data Availability

Not applicable.

## Abbreviations

WAO	Waste amine-oxime resin
FAO	Fresh amine oxime resin
MT-WAO	Waste amine-oxime resin after microwave pretreatment
FT-IR	Fourier-transform infrared spectroscopy
SEM-EDS	Scanning electron microscopy with energy dispersive spectroscopy
XPS	X-ray photoelectron spectroscopy
ICP-AES	Inductively coupled plasma-atomic emission spectrometry
TG-DTA	Thermogravimetry and differential thermal analysis
*K_d_*	distribution coefficient (Ratio of resin metal concentration to solution metal concentration at equilibrium, *K_d_* = *Qe*/*Ce* or *K_d_* = [(*C*_0_ − *Cs*)/*Cs*] × (*V*/*m*).
